# Supplementary material for: The interactome of intact mitochondria by cross-linking mass spectrometry provides evidence for coexisting respiratory supercomplexes
Source: Mol Cell Proteomics. 2017 Dec 8;17(2):216–32. doi: 10.1074/mcp.RA117.000470 (PMC5795388; doi:10.1074/mcp.RA117.000470)
Supplement: Supplemental Data [file supp_RA117.000470_133922_0_supp_23978_fzffwf.zip › spectra_annotation/mito_DR_spectra_annotation/40-1-4-1-10-1.pdf]

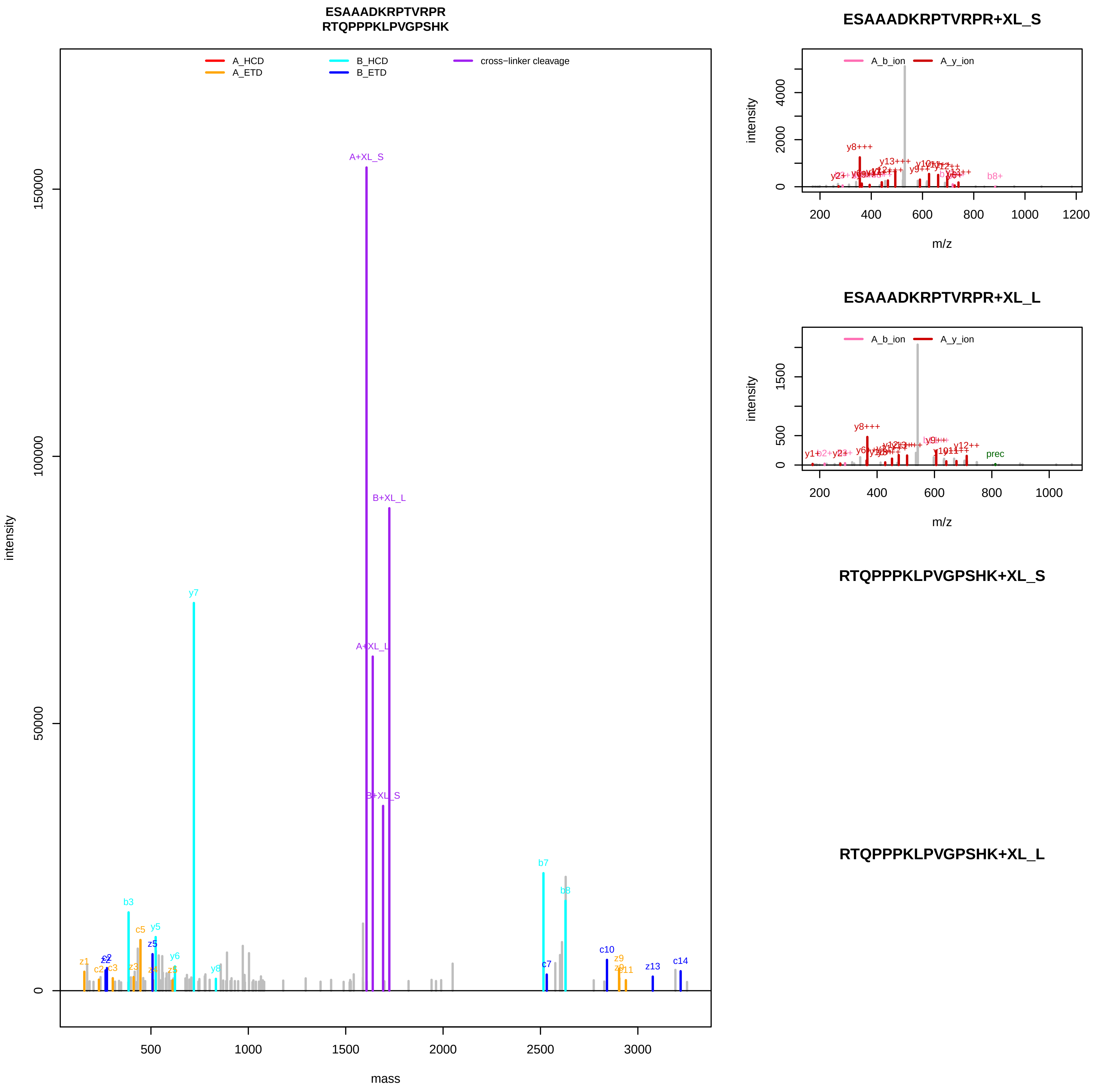
ESAAADKRPTVRPR
RTQPPPKLPVGPSHK
A_HCD
A_ETD
B_HCD
B_ETD
cross−linker cleavage
A+XL_S
B+XL_L
A+XL_L
B+XL_S
y7
b3
y5
c5
z5
y6
z1
c2
c3
z3
z4
y8
b7
b8
c7
c10
z9
z11
z13
c14
intensity
mass
0
50000
100000
150000
500
1000
1500
2000
2500
3000
ESAAADKRPTVRPR+XL_S
A_b_ion
A_y_ion
y8+++
y13+++
b8+
intensity
m/z
0
2000
4000
200
400
600
800
1000
1200
ESAAADKRPTVRPR+XL_L
A_b_ion
A_y_ion
y8+++
y12++
prec
y1+
intensity
m/z
0
500
1500
200
400
600
800
1000
RTQPPPKLPVGPSHK+XL_S
RTQPPPKLPVGPSHK+XL_L
